# Older people with HIV are an essential part of the continuum of HIV care

**DOI:** 10.1002/jia2.25188

**Published:** 2018-10-10

**Authors:** Eugenia L Siegler, Chelsie O Burchett, Marshall J Glesby

**Affiliations:** ^1^ Division of Geriatrics and Palliative Medicine Weill Cornell Medicine New York NY USA; ^2^ Division of Infectious Diseases Weill Cornell Medicine New York NY USA

**Keywords:** HIV care continuum, long‐term survivors, ageing, geriatric, differentiated care, quality of life, stigma

Advances in antiretroviral therapy (ARV) have offered people with HIV vastly improved, although not normal, life expectancies 1 and have changed the epidemiology of HIV care 2. Nonetheless, even though this is a worldwide phenomenon 3, with an estimated 6.7 million people 50 years and older living with HIV 4, we do not know how best to care for this population. Older people living with HIV (PLWH) are at higher risk than their uninfected counterparts for multimorbidity 5, cognitive impairment 6, polypharmacy 7, depression 8, loneliness 9, frailty 10 and other medical and social problems. To the already complex intersectionality of stigmas due to their HIV infection and to any of a combination of sexual orientation, gender identity, racism and injection drug use, one must add ageism 11, which can isolate older PLWH from groups that had been more welcoming when they were younger. Those of us who provide care to older PLWH must compete with other HIV constituencies for very limited funding to improve access and provision of care, while at the same time trying to determine best practices to meet the needs of this older, diverse population.

Appropriate care of older PLWH is a challenge in both resource‐rich and resource‐limited settings. In some areas, too much care – too many specialists, too many drugs – may be the primary problem. In other settings, even underserved areas within resource‐rich countries, lack of access to care (especially from subspecialties) because of inadequate resources, poor or no insurance, and/or stigma may be the primary concern. All settings require better integration of primary and specialty care; of mental health and medical health; and of medical setting and community. All settings must plan for burgeoning long‐term care needs.

Even in the absence of an evidence base for how care should be provided to older PLWH, clinical programmes have been initiated to try to address ageing issues. Table 1 provides examples of the basic models of care.

**Table 1:** Examples of care models for older people with HIV

Model	Location	Clinic/name	Venue	Comments
Geriatric consultation	Boston (United States)	Massachusetts General Hospital/Aging Positively 12	Biweekly in ID clinic	Providers may refer anyone older than 50. NP sees patients; develops plan with rest of team
	Brighton (England)	Brighton and Sussex University Hospital Silver Clinic 13	Monthly clinic sessions	Referral criteria: age >50, difficulty in coping at home, multimorbidity, polypharmacy; staff include HIV MD, geriatrician, HIV clinical nurse specialist, pharmacist
	Denver (United States)	University of Colorado	Outside consultation	Geriatrician, pharmacist see complicated older PLWH 1 to 3 times – refer back to primary care
	London (England)	Chelsea and Westminster Hospital 14	Separate multidisciplinary clinic	Referral criterion: age ≥ 50. Consultant, HIV NP, trainee; supported by specialist pharmacist and dietician
	Madrid (Spain)	Infanta Leonor University Hospital	In HIV clinic	Referral criteria: age ≥ 50; geriatrician does CGA for all, intervening as needed; yearly follow‐up for stable patients. Biweekly meetings with HIV providers
	Montreal (Canada)	McGill University	In HIV Clinic	Geriatrician sees referrals as needed; programme is planning for pharmacist, CGA for age >60
	New York (United States)	Weill Cornell Medicine/New York Presbyterian Hospital 15	Geriatrician weekly session w/in HIV clinic	No fixed referral criteria. Geriatrician follows longitudinally. Sponsors: arts, support groups, staff in‐services
	Salem, Virginia (United States)	SAVI	Veterans Affairs Hospital clinic	Assess multimorbidity, sarcopenia, frailty, cognition; Staff: pharmacist, neuropsychologist, dietician, endocrinologist
	San Francisco (United States)	Ward 86/Golden Compass 16	Geriatric HIV clinic: pharmacist, screen, geriatric consult	Opt‐out referrals for age >70, falls; Navigation is primary theme: heart/mind; strength/bones; screening/link to dental, vision, etc.; social work, CBSS, support groups
Metabolic Clinics			Age‐related assessments are part of the clinical programmes	These have a multimorbidity focus, but have evolved to incorporate evaluation of age‐related concerns like frailty and sarcopenia
	Melbourne (Australia)	Alfred Hospital/Monash University		
	Hong Kong SAR (China)	Prince of Whales Hospital, Chinese University of Hong Kong		
	Modena (Italy)	University of Modena and Reggio Emilia		
Online supports	Modena (Italy); Barcelona (Spain); Sydney (Australia) 17	Mysmartage.org	Smartphone app to assess and promote health	
	Tours (France)	Virtual passport	Online personal suggestions based on French guidelines for care of older PLWH	

Information from programmes that are not referenced was gleaned via personal communications. Readers wishing more information can contact the authors. CBSS, community‐based supports and services; CGA, comprehensive geriatric assessment; ID, infectious disease; NP, nurse practitioner; PLWH, people living with HIV.

One model for geriatric HIV programmes is consultative 16, where HIV providers can refer patients to ageing specialists, usually although not always geriatricians. These services may consist of a single geriatrician or a team of providers; may be embedded or external to the programme; may have specific or general referral criteria; and often include other services or programmes or linkages to community‐based organizations. The foundation of the consultation is comprehensive geriatric assessment (CGA), which encompasses medical, social, functional, psychological, and other domains 16. The advantages of this model are the ageing specialists’ understanding of the older person and the holistic frame of reference that CGA provides. The disadvantages are that (1) these ageing experts may not have effectively adapted tools designed for octo‐ and nonagenarians in the general population to PLWH, who may be several decades younger; and (2) as outsiders, consultants have little control over the implementation of their recommendations.

A second model has evolved from long‐standing metabolic clinical programmes that have provided care for and often studied PLWH with complex comorbidity and metabolic complications. Their providers have recognized the increasing prevalence of frailty and sarcopenia and are now trying to address them, as well. These programmes have the distinct advantage of having incorporated and internalized these age‐related concerns; treatment protocols are not recommended – they are implemented. The disadvantage of these programmes stems from their origins as metabolic clinics. Without vigilance, they run the risk of focusing too much on multimorbidity (especially if care of individual comorbidities is protocol‐driven) at the expense of psychosocial needs.

Some programmes have been initiated outside the clinical setting and are focusing on improving older PLWH engagement. One example is mysmartage.org, a smartphone platform that collects passively and actively inputted health information and offers coaching to help people stay healthy 17. We have also learned of a virtual passport in France to help older PLWH and their providers know what screening and preventive strategies are appropriate for them.

In addition to those listed in the table, many clinical programmes for older PLWH are in the planning stages or just starting. Through personal communications, we have learned of programmes both in the US (e.g. Philadelphia, Pennsylvania; San Diego, California; Durham, North Carolina) and other countries (e.g. Barcelona, Spain; Mexico City, Mexico; Porto Allegre, Brazil). In some of these nascent programmes, prospective cohorts specific to individual regions are shedding light on age‐related problems in local populations and some are giving rise to screening protocols for older PLWH. This is an exciting prospect; screening can be focused on particular aspects of geriatric care such as functional assessment of basic and instrumental activities of daily living 18, cognitive assessment with tools such as the Montreal Cognitive Assessment 19 or depression screening with tools such as the Patient Health Questionnaire‐9 20. Clinical staff can implement simple assessment tools with few added costs, but as with any form of screening, if there is no intervention available to those with impairments, screening may have limited value or may even be counterproductive.

Irrespective of venue, barriers to implementation of geriatric programmes include limited financing and insufficient buy‐in from leadership, lack of local geriatric expertise, inadequate social service resources and limited connections to the community. Although there is a robust literature about geriatric conditions, as of yet, there is no research base and no consensus about practice implementation: who should be targeted, which outcomes should be studied and how to measure success.

It is time to start generating that aforementioned evidence base. Local governments, insurers, and foundations must support efforts to create and test primary care innovations for older PLWH, whose input is essential to the design of geriatric HIV programmes. Programmes must not only offer medical care but also address the isolation and functional impairments of their constituents through integrated social programming 21.

Those in resource‐rich settings have much to share about management of comorbidities and polypharmacy, as well as CGA, with its prioritization of function and quality of life. People in resource‐limited settings with experience in differentiated services delivery 22 can share their experiences in creating connections to communities, reaching older PLWH where they are, engaging them and giving them more control over their care. These best practices should be collected through a global clearinghouse hosted by an HIV/AIDS organization with the ability to act as a resource hub for this information.

A recent editorial recommends that HIV care should be viewed as part of global health 23. In the same way, HIV care must be global and inclusive, embracing geriatric and palliative care so that older people can take advantage of the many years that successful antiretroviral therapy has enabled them to live.

## Competing interests

Drs. Siegler and Glesby and Ms. Burchett have support through their institution from an investigator‐initiated study funded by Gilead Sciences, Inc.

## Authors’ contributions

ES wrote the first draft of this Viewpoint. All authors contributed substantively to the writing of this manuscript and approved the final version.
